# Rigid Polyurethane Foams as Thermal Insulation Material from Novel Suberinic Acid-Based Polyols

**DOI:** 10.3390/polym15143124

**Published:** 2023-07-22

**Authors:** Aiga Ivdre, Arnis Abolins, Nikita Volkovs, Laima Vevere, Aigars Paze, Raimonds Makars, Daniela Godina, Janis Rizikovs

**Affiliations:** 1 Latvian State Institute of Wood Chemistry, 27 Dzerbenes St., LV-1006 Riga, Latvia; 2 PolyLabs SIA, 46 Mukusalas St., LV-1004 Riga, Latvia

**Keywords:** polyurethane, rigid polyurethane foams, suberin, bio-polyols, suberinic acids, sustainability, renewable materials

## Abstract

Developing polyols from biomass sources contributes to a more circular economy by replacing petroleum-based polyols in the vast production of polyurethanes (PUR). One such potential biomass source could be leftover birch bark from which suberinic acids (SA) can be obtained. The purpose of this study was to identify the best synthesis routes for novel SA-based polyols, obtain rigid PUR foams, and evaluate their competitiveness and potential suitability as thermal insulation material. Novel polyols were synthesized from depolymerized SA by esterification with various functionality and molecular weight alcohols in several molar ratios. The moisture content, hydroxyl and acid values, and apparent viscosity were tested. Free-rise rigid PUR foams from the most suitable SA-based polyol and tall oil-based polyol were successfully prepared, reaching ~20 wt.% total renewable material content in the foam. The obtained rigid PUR foams’ morphological, mechanical, and thermal properties were investigated and compared to present foam materials, including commercial foams. The apparent density (~33 kg/m^3^), as well as the closed cell content (~94%), compression strength (0.25 MPa, parallel to the foaming direction), and thermal conductivity (~0.019 W/(m·K)), approved the competitiveness and potential ability of SA-based rigid PUR foam production as thermal insulation material.

## 1. Introduction

Sustainability has become one of the most essential topics in the world right now, driven by global climate change and the shortage of petrochemical resources. The replacement of fossil materials by expanding the utilization of renewable biomass, recycled, and waste resources, is crucial to the circular bio-economy’s efforts to advance sustainability [1,2]. Plastic materials are widely used and manufactured mainly from fossil fuels, such as coal, natural gas, or crude oil [3].

The sixth most popular form of polymer used globally is polyurethane (PUR). It is a highly versatile material applied as coatings, adhesives, sealants, elastomers, and flexible and rigid foams. Rigid PUR foams are commonly used in buildings’ thermal insulation and commercial refrigeration [4]. Polyols, one of the primary basic components for PUR production, are synthesized mainly from fossil-based materials. Bio-based alternatives have been widely studied, starting with natural oils competing with the food industry (the first generation of biomass feedstock) and ending with genetically modified seeds [5], and genetically engineered algae [6] (the fourth generation of biomass feedstock). The second generation includes inedible oils, wood biomass derivatives, and waste products [7]. Inedible oils solved the fundamental issue of the first generation by eliminating direct competition in food and livestock feed, but agricultural land remained the competitive element. Hence, it is crucial to exploit low-value waste biomass streams.

Many companies, including those producing furniture, veneer, and cellulose, incinerate a significant amount of leftover birch bark for energy production [8]. Processing birch bark offers the potential to obtain more products with higher added value, such as triterpene-rich extracts containing betulin, lupeol [9], and depolymerized suberinic acids (SA) [8]. The most prevalent category of suberin monomers in the outer bark of *Betula pendula* typically is ω-hydroxy fatty acids. The primary components of the depolymerized SA mixture are epoxy and hydroxyl groups that contain monomers and oligomers [10,11]. Only a few studies have been published where rigid PUR foams were obtained from SA-based polyols. Depolymerized SA from birch outer bark has been investigated and presented as an alternative resource to develop bio-based polyols via the esterification reaction with diethylene glycol (DEG) [8]. In addition, the possibility of obtaining rigid PUR foams from polyols synthesized by SA esterification with triethanolamine (TEOA) has been reported. The obtained rigid PUR foams showed good mechanical properties and reached total renewable material content of 16.6% [12]. These first few investigations have revealed and proved the potential of *Betula pendula* SA application in polyol synthesis suitable for rigid PUR foams development. However, more studies are crucial to compare different polyols and find appropriate rigid PUR foam formulations to obtain materials with properties similar to or better than commercial products.

The purpose of this study was to identify the best synthesis routes for novel SA-based polyols, obtain rigid PUR foams, and evaluate their competitiveness and potential ability as thermal insulation material. This work aimed to synthesize SA-based polyols by SA esterification with six different multifunctional alcohols, choose the most appropriate polyols, and obtain rigid PUR foams. The acid, hydroxyl value, and viscosity of the polyols were evaluated for application in producing rigid PUR foams. The rigid PUR foams were characterized with relevant properties for thermal insulation material and compared with reference foams from other investigations in the literature, including a commercial product.

## 2. Materials and Methods

### 2.1. Materials for Polyol Synthesis and Analysis

The SA mixture originates from wood biomass after depolymerizing birch outer bark, which was kindly supplied by AS Latvijas Finieris plywood factory (Latvia) as industrial waste. The SA mixture with an acid value of 90 mg KOH/g, a hydroxyl value of 120 mg KOH/g, and an apparent viscosity of 6,09·10^6^ mPa·s at the shear rate 50 s^−1^ was prepared by our research team at the Latvian State Institute of Wood Chemistry, as described by Rizikovs et al. [8]. In addition, 1,4-butanediol (BD), reagent plus, ≥99%; ethylene glycol (EG) puriss p.a., 99.0%; diethylene glycol (DEG), puriss p.a., 99.0%; diethanolamine (DEOA), reagent grade, ≥98.0%; triethanolamine (TEOA), reagent grade, 98.0%; and trimethylolpropane (TMP), reagent grade, 97%; acetic anhydride, puriss, ≥99%; 4-(dimethylamino)pyridine, reagent plus, ≥99%; methanol, puriss p.a., ACS reagent, ≥99.8%; N, N-dimethylformamide, ACS reagent, ≥99.8%, water content ≤150 ppm; potassium hydroxide (KOH), ACS reagent, pellets, ≥85%, were purchased from Sigma Aldrich.

### 2.2. Materials for Rigid PUR Foam Production

For the production of rigid PUR foams the following materials were used as purchased: tris(1-chloro-2-propyl)phosphate (TCPP) as a flame retardant (Albermarle, Louvain-la-Neuve, Belgium); tertiary amine-based catalyst PC CAT NP10^®^ and 30 wt.% of potassium acetate in DEG PC CAT TKA 30^®^ (Air Products and Chemicals Inc., Rotterdam, The Netherlands); and Niax Silicone L-6915 as a surfactant (Momentive Performance Materials Inc., Hamburg, Germany). Opteon™ 1100 (Chemours, Dordrecht, The Netherlands) was used as a low global warming potential physical blowing agent. Additionally, distilled water was added as a chemical blowing agent. Bio-polyol from epoxidized tall oil and trimethylolpropane (ETO_TMP) was synthesized as previously published (OH = 460 mg KOH/g, H_2_O = 0.11%, renewable material content 63.2%) (Latvian State Institute of Wood Chemistry, Riga, Latvia) [13,14]. Desmodur 44V20 L (pMDI) was used as the isocyanate component (Covestro, Leverkusen, Germany). It is a solvent-free product based on 4,4′-diphenylmethane diisocyanate and contains oligomers with high functionality. The average functionality of pMDI is 2.8–2.9, and the NCO group content is 31.5 wt.%.

### 2.3. Synthesis of SA-Based Polyols

To obtain SA-based polyols, the esterification reaction was carried out in a four-neck round bottom flask to which the appropriate mass of SA (100 g), KOH as a catalyst (0.5 wt.% of SA), and the multifunctional alcohol was added; see Table 1 for corresponding alcohol mass for each polyol.

The molar amount of each multifunctional alcohol needed for bio-polyol synthesis (Table 1) was calculated using the following Equation (1):

n_(AV)_ = n_(alc)_, (1)

where n_(AV)_ is the molar amount of the SA acid value in mol, and n_(alc)_ is the molar amount of the alcohol used for the esterification reaction in mol. The flask was immersed in an oil thermostatic bath, and a mechanical stirrer was inserted into the central neck. A purge gas tube, a Liebig condenser, and a thermometer were attached to the vacant necks. The mechanical stirrer was set to 450 rpm.

**Figure 1:**
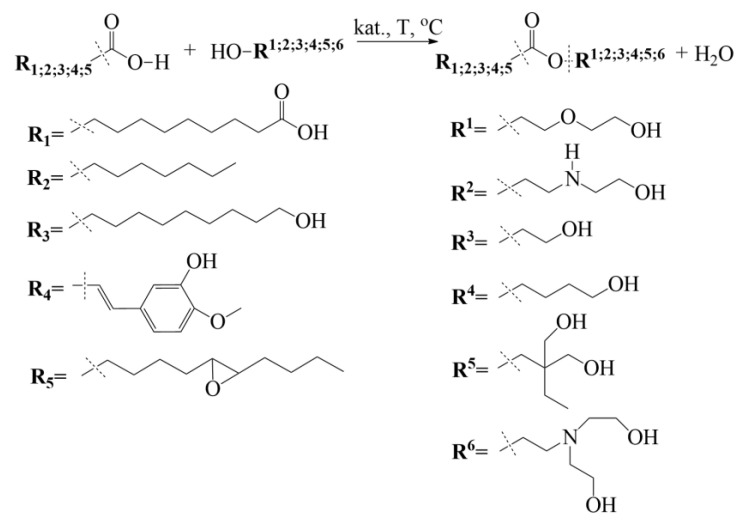
Idealized esterification reaction of the SA with multifunctional alcohols.

The synthesis temperature for each used multifunctional alcohol to carry out the esterification reaction can be seen in Table 1. The stirring of the reaction medium at the indicated temperature and gas flow was retained for 6 h for each synthesized polyol. The samples were taken each hour to evaluate the acid value. After synthesis, the obtained bio-polyol was analyzed for the hydroxyl value and apparent viscosity to assess suitability for rigid PUR foam development. The theoretical esterification reaction scheme of the SA with multifunctional alcohols is shown in Figure 1.

### 2.4. Characterization of the Synthesized SA-Based Polyols

#### 2.4.1. Acid Value

The acid value was determined according to the ISO 2114:2000 standard [15].

#### 2.4.2. Hydroxyl Value

The hydroxyl value was determined according to the ISO 4629-2:2016 standard [16].

#### 2.4.3. Moisture Content

The moisture content was measured using Karl Fischer titration, using the Denver Instrument Model 275KF automatic titrator (Denver Instrument, New York, NY, USA).

#### 2.4.4. Apparent Viscosity

The rheological measurements were made using an Anton Paar modular compact rheometer MCR 92 (Anton Paar, Graz, Austria), with a cone-plate measuring system and a gap of 48 μm. The apparent viscosity of the polyols was measured at 25 °C and the shear rate was 50 s^−1^ using standard flow curve measurement and shear rate sweep from 0.1 s^−1^ to 100 s^−1^.

#### 2.4.5. Fourier Transform Infrared Spectrometry

The polyol structure was analyzed using Fourier transform infrared spectrometry data (FTIR), which were obtained with a Thermo Scientific Nicolet iS50 spectrometer (Thermo Fisher Scientific, Waltham, MA, USA) at a resolution of 4 cm^−1^ (32 scans). The FTIR data were collected using an attenuated total reflectance technique with ZnSe and diamond crystals.

### 2.5. Rigid PUR Foam Preparation and Formulation

The free-rise rigid PUR foams were prepared, according to the formulations reported in Table 2, by mixing the polyol and polyisocyanate components. An NCO/OH ratio of 1.2 was chosen. Each of the three types of SA-based polyols (SA_TEOA_2, SA_TMP_2, SA_BD_2) was used as the main polyols in five rigid PUR foam formulations. Our unpublished preliminary experiments revealed that an SA-based polyol amount higher than 70 wt.% resulted in ineffective mixing and caused flaws in the structure of the foams. Hence, the main polyols were added in two concentrations: 70 wt.% and 50 wt.% of the total polyol amount. Water in the polyols was considered, and the amount of added distilled water was adjusted to achieve the exact value of total water (2 g) in all formulations. At first, all the polyol components, except the SA-based polyol and physical blowing agent, were weighed and mixed in a plastic cup using a mechanical stirrer for ~1 min at 2000 rpm. Then, the SA-based polyol (preheated to 90 °C to decrease the viscosity and enhance the mixing quality) was added and remixed for ~2 min. Opteon™ 1100 was added as the last ingredient in the polyol component and mixed for ~1 min. When the polyol component was cooled to room temperature (22 °C, measured with a Greisinger GMH 3700 Series Pt100 thermometer), the isocyanate component, namely pMDI, was added and mixed for 10 s. Afterwards, the mixture was instantaneously poured into an open mold with the dimensions 30 × 30 × 10 cm to obtain free-rise foams. All the foam samples were allowed to cure at ambient conditions for 24 h before any further tests.

The synthesized PUR foams were named as follows: ALCOHOL_nn, where ALCOHOL corresponds to the multifunctional alcohol, namely the esterification reagent for the primary polyol synthesis (TEOA, TMP, or BD refers to the polyols SA_TEOA_2, SA_TMP_2, and SA_BD_2, respectively), and nn corresponds to the polyol’s percentage of the total polyol mass, 70 wt.% or 50 wt.%.

### 2.6. Rigid PUR Foam Characterization

The prepared rigid PUR foam blocks samples for various tests were cut with a band saw. The foam quality was visually assessed, and images of the foam samples were captured by a camera.

#### 2.6.1. Foaming Parameters

The foaming parameters start time and rise time, as well as the shrinkage after 24 h, were measured with the universal foam qualification system Foamat. A glass rod was repeatedly dipped and pulled out of the reaction mixture to measure the gel time (also known as the string time). The gel time was reached when the strings appeared after removing the rod from the reaction mixture. Afterwards, the surface of the foam was touched gently with the glass rod several times. When no foam adhered to the rod, it was noted as tack-free time.

#### 2.6.2. Apparent Density

The apparent density of the obtained PUR foams was tested according to the ISO 845:2006 standard [17].

#### 2.6.3. Thermal Conductivity

The thermal conductivity coefficient (λ) was tested with a FOX 200 by TA instruments-Water LLC, according to the ISO 8301:1991 standard [18], at an average temperature of 10 °C (cold plate: 0 °C, and hot plate: +20 °C, sample dimensions: 200 × 200 × 30 mm). 

#### 2.6.4. Closed Cell Content

The closed cell content was measured and calculated, according to ISO 4590:2016 [19], with a pycnometer AccuPyc II 1340 for specimens with dimensions 30 × 30 × 55 mm. 

#### 2.6.5. Cell Size

Slices of foam, as thin as possible, were cut with a razor blade parallel and perpendicular to the foam rise direction. A light microscope, Diamond MCXMP500, MICROS Produktions-&Handels GmbH, Austria, was used to investigate the PUR foams’ cellular structure at a magnification of 10 times. The obtained images were processed with ImageJ software, and the average cell diameter parallel (direction Z) and perpendicular (direction X) to the foam rise direction was measured according to the ASTM D 3576 standard [20]. It was assumed that there was no measurable edge-to-edge or top-to-bottom variation in the average cell size and that the cell size distribution about the average cell size was normal. Geometrical anisotropy was calculated by dividing the average cell diameter Z by the average cell diameter X.

#### 2.6.6. Compressive Strength and Modulus

The compressive strength and modulus parallel and perpendicular to the foam rise direction were tested, according to the requirements of the ISO 844:2021 standard [21], on a testing machine Zwick/Roell Z100 (maximum load-cell capacity 1 kN, the deformation rate: 10%/min) for cylinder specimens with a diameter and height of ~20 mm. These cylindrical samples were cut with a drill press using a crown drill bit. Six specimens were analyzed for each PUR foam formulation, and the average value was taken along with the standard deviation. The compression strength and modulus results were normalized to a unified apparent density (40 kg/m^3^), according to Hawkins et al. [22], to obtain comparable results.

#### 2.6.7. Sample Grounding

The rigid PUR foam samples were ground into a fine powder using a ball mill from Retsch GmbH CryoMill at liquid nitrogen temperature (−196 °C). A zirconium oxide milling vessel with six 10 mm zirconium oxide balls was used to obtain the PUR powder. The following milling procedure was used: precooling to a temperature of −196 °C for 15 min at a vibrational frequency of 5 Hz, then six cycles of cryogenic milling for 5 min at 25 Hz vibrational frequency, and milling for 40 min at 30 Hz vibrational frequency until the sample heats to room temperature.

#### 2.6.8. FTIR Spectrometry

The grounded samples of rigid PUR foam were analyzed by FTIR spectrometry in the same way as the polyols, namely using a Thermo Scientific Nicolet iS50 spectrometer at a resolution of 4 cm^−1^, 32 scans. The FTIR data were collected using an attenuated total reflectance technique with ZnSe and diamond crystals.

#### 2.6.9. Differential Scanning Calorimetry (DSC)

DSC was performed on a TA Instrument DSC Q1000 under a nitrogen atmosphere, using approximately 5 mg of each grounded sample. The samples were initially heated from ambient temperature to 170 °C at a heating rate of 10 °C/min, cooling until −50 °C and, then, heated again at a rate of 10 °C/min to 170 °C. The preheating step (from ambient temperature to 170 °C) was conducted to remove the nonreversible thermal effects like releasing internal stress, the change of macromolecular conformation, and crosslinking due to traces of unreacted isocyanate. A second scan was used to determine the glass transition temperature.

#### 2.6.10. Dynamical Mechanical Analysis (DMA)

The DMA was carried out with a Mettler Toledo DMA/SDTA861e with the following parameters: a temperature range from −100 °C to 210 °C, a ramp rate of 3 °C/min, a frequency of 1 Hz, an amplitude of 5 μm, and a maximal force of 1 N. The compression oscillation mode was used. The rigid PUR foam samples with a diameter of ~13 mm and a height of ~7 mm were used for the tests. Smoothing with the adjacent-averaging method was conducted since the curves after 170 °C were noisy.

#### 2.6.11. Thermogravimetry Analysis (TGA)

The samples were analyzed using a TA Instruments Discovery TGA thermogravimetric analyzer and autosampler. The grounded PUR foam samples were placed on platinum scale pans and heated in a nitrogen atmosphere at 10 °C/min in a temperature range between 30–700 °C. At least three parallel samples of fine PUR powder were tested and analyzed. The data were processed using the OriginPro 2021 9.8.0.200 and TA Instruments TRIOS #5.0.0.44608 software.

#### 2.6.12. SA and Total Renewable Material Content

In addition, the SA and total renewable material content were calculated by the SA mass or total mass of the renewable feedstock (SA, water, tall oil) in the rigid PUR foam formulation divided by the total mass of the PUR foam. The result was expressed in weight percentage (wt.%).

## 3. Results and Discussion

### 3.1. Polyol Characterization

The SA was used as a raw material for synthesizing twelve bio-polyols. The esterification of the acid carboxyl groups was conducted with various functionality and molecular weight alcohols, such as BD, EG, DEG, DEOA, TEOA, and TMP. KOH was used as an esterification catalyst. The overall synthesis temperatures ranged from 170 °C to 185 °C except for the SA polyols synthesized from DEOA. The experience of our research team has shown that when the temperature exceeds 150 °C, the DEOA tends to undergo cyclization reactions. Hence, the temperature of 145 °C for the polyol synthesis with DEOA was chosen to avoid undesirable reactions. Polyols synthesis by tall oil fatty acids esterification with DEOA at 145 °C has been documented [23,24]. Rapeseed oil transesterification with TEOA has been reported at 170 ± 5 °C temperature [25]; also, for tall oil fatty acids transesterification with TEOA a similar synthesis temperature has been chosen (173 ± 3 °C) [26]. Our preliminary experiments showed that the optimal synthesis temperature for SA esterification with BD, EG, DEG, and TEOA is between 170 °C and 200 °C.

Table 3 summarizes the typical characteristics of synthesized SA-based polyols, including the acid value, hydroxyl value, viscosity, moisture content, and renewable content. The SA-based polyols are investigated and discussed below for suitability and application in rigid PUR foam development.

#### 3.1.1. Acid Value

**Figure 2:**
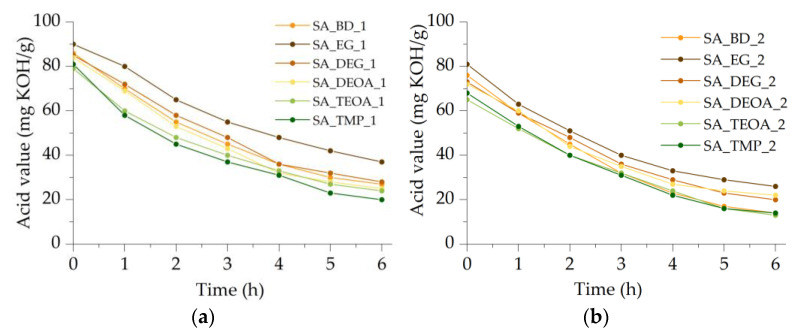
The acid value kinetics during the esterification reaction for: (**a**) the polyol synthesis with a molar ratio of 1:1; (**b**) the polyol synthesis with a molar ratio of 1:2.

This study focused on synthesizing SA-based polyols using SA as the raw material. A total of twelve polyols were prepared, with six synthesized using a molar ratio of 1:1 (free carboxyl group to alcohol), and the remaining six using a molar ratio of 1:2. The acid values of the synthesized polyols showed a wide range, ranging from 13 mg KOH/g to 37 mg KOH/g. The acid value kinetics during the esterification reaction are depicted in Figure 2a,b.

Figure 2a illustrates the results when a 1:1 molar ratio was used, revealing that the acid values of the synthesized polyols did not drop below 20 mg KOH/g. Among the polyols synthesized using a molar ratio of 1:1, the highest acid value of 37 mg KOH/g was observed for the SA-based polyol synthesized from EG, while the lowest acid value of 20 mg KOH/g was achieved when TMP was used as the esterification reagent. A noticeable pattern emerged, indicating that when bifunctional alcohols with a lower molecular weight were employed, the decrease in acid value was slower, resulting in slightly higher acid values in the final products compared to synthesis with trifunctional alcohols. This trend persisted when a molar ratio of 1:2 was employed. Most bifunctional alcohols, such as EG, DEG, and DEOA, yielded polyols with acid values not dropping below 20 mg KOH/g during the esterification of SA. Notably, when DEOA was used as the alcohol, an amidation reaction could also occur. Furthermore, when a molar ratio of 1:2 was used, the SA-based polyols synthesized using BD or trifunctional alcohols like TEOA or TMP resulted in acid values of 14 mg KOH/g after a 6 h esterification reaction.

Overall, when a 1:2 molar ratio was employed, the resulting polyols exhibited lower acid values compared to a molar ratio of 1:1. However, it is essential to consider that increasing the amount of multifunctional alcohol also influenced other properties, such as the renewable material content, hydroxyl value, and viscosity.

In industrial applications, it is well-known that polyols intended for rigid PUR foam production should have low acid values. The literature reports polyol synthesis with acid values below 10 mg KOH/g [14], approximately 15 mg KOH/g [15], or even 25 mg KOH/g [16]. High acid values in polyols can lead to unnecessary increases in the quantity of the catalyst required for preparing PUR systems and can adversely affect the stability and quality of the resulting rigid PUR foams. Therefore, this study selected three SA-based polyols with acid values lower than 15 mg KOH/g for experimental rigid PUR foam production.

#### 3.1.2. Viscosity

**Figure 3:**
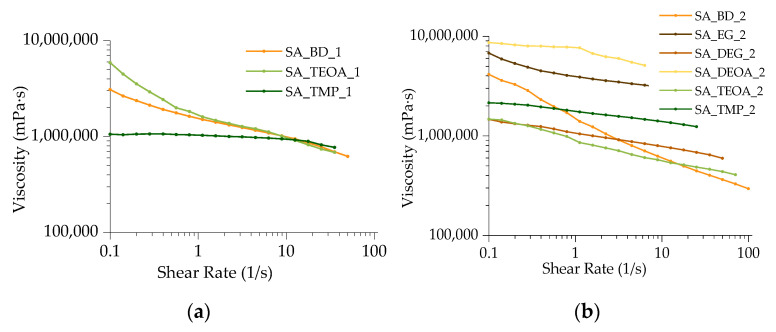
The rheological measurements of SA-based polyols: (**a**) synthesized using the carboxyl acid group to alcohol molar ratio of 1:1; (**b**) synthesized using the carboxyl acid group to alcohol molar ratio of 1:2.

Viscosity measurements were conducted on nine SA-based bio-polyols, utilizing molar ratios of carboxyl groups to alcohol of either 1:1 or 1:2. Among the polyols synthesized with a 1:1 ratio, only three were evaluated for viscosity properties because polyols SA_EG_1, SA_DEG_1, and SA_DEOA_1 exhibited extremely high viscosities and poor flow properties, solidifying upon cooling and making their recovery from the flask impossible. Figure 3a illustrates the rheological properties of three polyols synthesized with a 1:1 ratio, while Figure 3b displays the results for six polyols synthesized with a 1:2 ratio. All the SA-based polyols exhibited non-Newtonian behavior, characterized by pseudoplastic flow, where an increase in the shear rate led to a decrease in the viscosity within the range of 0.1 to 100 s^−1^. The flow curves of the polyols synthesized using both molar ratios displayed various behaviors. The polyols obtained from BD, TEOA, and TMP demonstrated flow properties, allowing recovery from the synthesis flask while still in a hot state. Nonetheless, when subjected to viscosity and flow tests at 25 °C, the SA-based polyols such as SA_BD_1, SA_TEOA_1, and SA_TMP_1 exhibited excessively high values, rendering them unsuitable for rigid PUR foam production. These polyols also displayed a shear rate-dependent viscosity ranging from 5.6·10^6^ mPa·s to 6.2·10^5^ mPa·s, surpassing the measurement apparatus’s capacity to register shear rates of 100 s^−1^. The viscosity dependence on the shear rate for the SA_BD_1, SA_TEOA_1, and SA_TMP_1 polyols ceased at 50.1 s^−1^, 35.5 s^−1^, and 35.5 s^−1^, respectively. Extrapolated values of apparent viscosity at a shear rate of 50 s^−1^ can be found in Table 3. Considering the viscosity properties and acid value, none of the SA-based polyols synthesized with a 1:1 ratio were suitable for PUR foam production.

Furthermore, the viscosity and flow properties of the SA-based polyols utilizing a 1:2 molar ratio are presented in Figure 3b. This ratio made it possible to recover all the synthesized polyols. However, not all the polyols reached a shear rate of 100 s^−1^ due to the apparatus’s inability to register high shear stress. In such cases, the apparent viscosity values were extrapolated and are provided in Table 3. The polyol with the highest viscosity was SA_DEOA_2, synthesized using DEOA as the alcohol, and its measurable flow properties were observed within the shear rate range of 0.1 s^−1^ to 6.3 s^−1^, with viscosity values ranging from 8.6·10^6^ mPa·s to 5.1·10^6^ mPa·s. The fastest decrease in viscosity was observed for SA_BD_2, where the viscosity decreased from 4.14·10^6^ mPa·s to 2.9·10^5^ mPa·s. Among the polyols, the one synthesized from BD exhibited the lowest apparent viscosity of 3.6·10^5^ mPa·s. The remarkably high viscosity of the SA-based polyols can be attributed to the presence of numerous molecular mass products and their highly branched structure. This high viscosity poses a challenge for the further advancement of the technology, as it restricts their use in processing equipment due to their specific requirements.

#### 3.1.3. Hydroxyl Value

The hydroxyl values of the synthesized SA-based polyols ranged from 304 mg KOH/g to 565 mg KOH/g. Notably, when employing a molar ratio of 1:1 (carboxyl acid group to alcohol), the bio-polyols exhibited lower hydroxyl values compared to those obtained with a molar ratio of 1:2. This disparity can be attributed to the excess alcohol used in the reaction mixture resulting in a higher concentration of unreacted alcohol, thereby increasing the hydroxyl value.

It is worth mentioning that for certain bio-polyols, namely SA_EG_1, SA_DEG_1, and SA_DEOA_1, the determination of the hydroxyl value was not conducted. This was due to the inherent limitations in recovering these products from the synthesis flask after completion. These specific polyols displayed inadequate flow properties, rendering their recovery impractical.

The hydroxyl values observed in the SA-based polyols, falling within the typical range for rigid PUR foam development, are noteworthy. However, considering other pivotal factors, such as the acid value and viscosity flow parameters, a substantial number of products did not meet the required criteria and were deemed unsuitable for rigid PUR foam production.

These findings underscore the significance of carefully evaluating multiple properties, including the hydroxyl value, acid value, and viscosity flow parameters, to ensure the suitability of SA-based polyols for application in rigid PUR foam formulations.

#### 3.1.4. Moisture Content

Moisture content is an important parameter to consider when synthesizing and applying polyols, especially in the context of rigid PUR foam development. The moisture content in polyols can significantly affect the final foam properties and processing characteristics.

All the SA-based polyols synthesized in this study exhibited moisture content levels well below 0.2%. Consequently, the moisture content was considered negligible concerning their suitability for developing rigid polyurethane PUR foams. For specific polyols, the determination of moisture content was omitted due to their unsuitability for rigid PUR foam preparation, primarily attributable to other critical properties such as the acid value and viscosity. Thus, the moisture content analysis was secondary in assessing the polyols’ applicability for rigid PUR foam production.

#### 3.1.5. Renewable Content

The renewable content of SA-based polyols was strongly influenced by the molar ratios of free carboxylic groups to alcohol and the molecular weight of the diol or triol used in the synthesis process. A higher molecular weight of the alcohol and a greater difference in the molar ratios resulted in lower renewable content. When the molar ratio of carboxylic groups to alcohol was 1:1, the renewable content ranged from 80.7% to 90.9%. However, when the ratio was increased to 1:2, the renewable content of the polyols decreased significantly, ranging from 67.6% to 83.4%. Additionally, the molecular weight of the alcohol played a crucial role in determining the renewable content. The polyols synthesized with ethylene glycol (EG), which has a molecular weight of 62.1 g/mol, exhibited the highest renewable content among all the molar ratios (1:1 and 1:2). Conversely, the polyols synthesized from triethanolamine (TEOA) with a molecular weight of 149.2 g/mol had the lowest renewable content.

### 3.2. Rigid PUR Foam Characterization

#### 3.2.1. Foam Morphology

**Figure 4:**
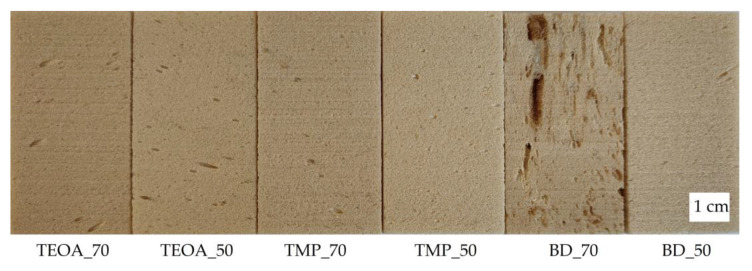
Rigid PUR foam samples’ visual appearance.

Six free-rise rigid PUR foam samples were prepared from the chosen most suitable three SA-based polyols (SA_TEOA_2, SA_TMP_2, and SA_BD_2), according to the PUR formulations reported in Table 2 in Section 2. The SA-based polyols were added in two percentages, namely 70 wt.% and 50 wt.% of the total polyols’ amount. The visually assessed quality of all the rigid PUR foam samples was acceptable except for sample BD_70. The obtained BD_70 foam was of poor quality, with many defects, namely large, deformed cells, cracks, and visible unreacted mix (Figure 4). The poor quality of the BD_70 foam could be attributed to insufficient mixing quality or incompatibility with the blowing agent because of the increased polyol content in the PUR formulation.

**Figure 5:**
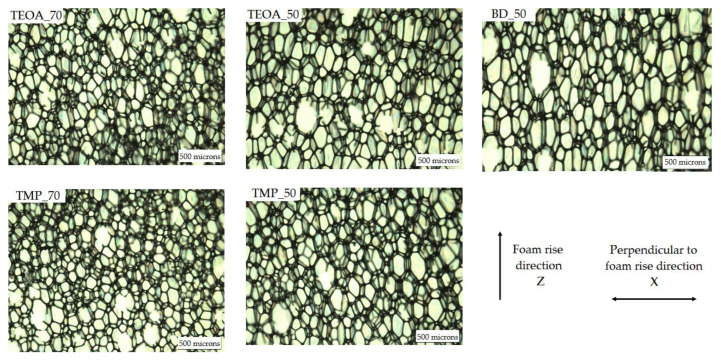
Optical microscope images of the rigid PUR samples.

The OM images of the rest of the developed rigid PUR samples, presented in Figure 5, reveal neat, elongated cell structures. The measured and calculated morphology parameters are shown in Table 4. The anisotropy index for the investigated rigid PUR foam samples varied from 1.2 to 1.6. Anisotropy occurs during rapid foam synthesis and is commonly observed in free-rise PUR foams [27].

Foams with lower SA-based polyol content showed larger cells, which was approved by the average cell size measurement results. The materials’ cellular structure is affected by various parameters, including the apparent density, reactivity, and viscosity [28]. The higher content of SA-based polyol increased the overall viscosity of the polyol component. The higher viscosity of the polyol component complicated the foaming step by slowing the foam formation [29]. It could contribute to less cell drainage by gravity during the initial foaming stage [30], resulting in a smaller average cell size. The average cell diameter in a parallel and perpendicular direction to the foam rise varied from ~235 µm and ~200 µm, respectively (TEOA_70 and TMP_70), to ~370 µm and ~235 µm, respectively (BD_50). The average cell size for low-density free-rise rigid PUR foams can reach almost 500 µm [31]. The smaller the cells, the better the foams’ mechanical properties (e.g., compression strength) [22]. That is why the reduction in the cell size of rigid PUR foams is viewed as a beneficial impact [32].

The closed cell content is one of the main characteristics of thermal insulation materials, which should be higher than 90%. All the developed rigid PUR foam samples except BD_70 met this criterion. Because of the BD_70 sample’s poor quality, further testing was not conducted. Therefore, this paper broadly characterizes the other five successful rigid PUR foam samples (TEOA_70, TEOA_50, TMP_70, TMP_50, and BD_50).

#### 3.2.2. Foaming Parameters and Basic Characteristics

A summary of the developed rigid PUR foams’ selected properties is presented in Table 5. The SA content in the PUR foams varied from 10.1% to 14.3%, where a maximum was reached for TEOA_70. The concentration of SA-based polyols did not significantly affect the total renewable material content because the second polyol (ETO_TMP) in the PUR formulation was also made from renewable materials. Hence, all the PUR samples had approximately the same amount of total renewable raw materials (~20%).

The foaming start time was affected mainly by the structure and amount of SA-based polyol and the concentration of the catalyst Polycat NP10. The PUR formulations based on the SA_TEOA_2 polyol required four times less foaming catalyst, as they showed higher reactivity. The autocatalytic properties of TEOA-type bio-polyols due to the tertiary amine group in the main polyol structure have been reported previously [13,33,34]. A higher amount of SA_TMP_2 polyol slows the foaming reaction (all the foaming parameters for TMP_70 were higher than for TMP_50) because of the higher viscosity of polyol.

Faster growth of the foam decreases the foam’s apparent density. Therefore, less blowing agent was added into TEOA_70 to obtain a similar apparent density for all the PUR samples. The obtained apparent density (32–35 kg/m^3^), as well as the closed cell content (93–95%), was in the range of the PUR foams traditionally used for thermal insulation in buildings (apparent density 30–45 kg/m^3^; closed cell content > 90%). The compression strength (0.24–0.27 MPa) was even more than two times higher than is sufficient for many rigid PUR foam applications [35,36]. All the rigid PUR foams’ samples showed excellent low thermal conductivity at ~0.019 W/(m·K). The base polyol type and cell size did not affect the thermal conductivity, as it depends mainly on the thermal conductivity of the gases trapped within the foam cells [37].

#### 3.2.3. Normalized Compressive Strength and Modulus

**Figure 6:**
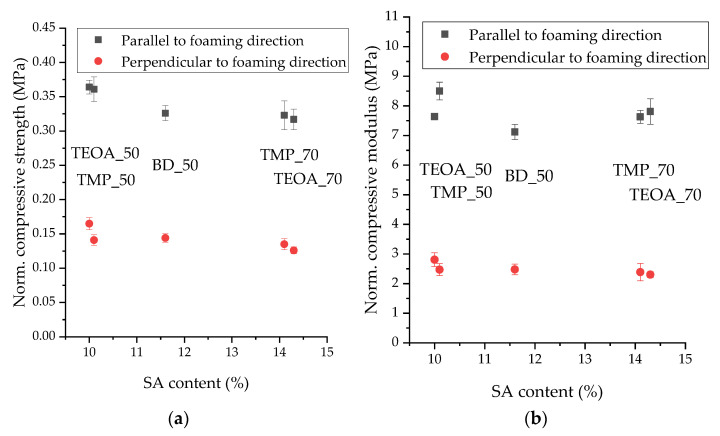
Physical–mechanical properties of the rigid PUR foams: (**a**) normalized compressive strength; (**b**) normalized compressive modulus.

The apparent density of the obtained rigid PUR samples slightly varied. Therefore, the compressive strength and modulus were normalized with respect to the apparent density of 40 kg/m^3^, using the equations by Hawkins et al. [22]. These results are shown in Figure 6, depending on the SA content in the foam. The names of the PUR samples are given in the graph to evaluate the impact of the polyol type on the compressive strength and modulus. Compared to the perpendicular direction, the compressive strength and modulus in the direction parallel to the foam rise were lower. The mechanical properties of the foams were anisotropic due to the geometrical anisotropy of the cells, which varied from 1.2 to 1.6. This anisotropy is often best represented by the transverse isotropy, with more strength and stiffness displayed in the rising direction with elongated cells [38]. A similar effect has been observed by other researchers too [27,39].

On the compressive strength and modulus results, several relationships were observed. The compressive strength slightly decreased as the SA content increased in both directions, namely parallel and perpendicular to the foam rise direction. The difference between the SA_TEOA and SA_TMP polyols did not affect the compressive strength of the respective rigid PUR foams (observed when comparing TEOA_50 with TMP_50, and TMP_70 with TEOA_70). Although the SA content differs, the compressive strength of BD_50 within margins of error coincides with the compressive strength of TMP_70 and TEOA_70. In addition, BD_50 showed the lowest compressive modulus parallel to the foam rise direction, possibly because it had the largest cell size. The compressive modulus of the TMP_50 sample was slightly higher than all the others, indicating a more remarkable ability to absorb energy.

#### 3.2.4. Thermal Characterization

**Figure 7:**
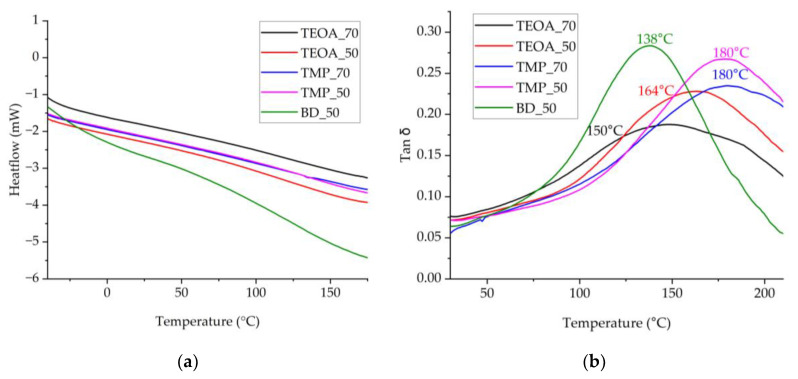
Detection of the glass transition temperature of the rigid PUR foams: (**a**) DSC curves, exo up; (**b**) damping factor tan δ of the DMA results.

DSC was performed in the temperature range from −50 °C to 170 °C to determine the glass transition temperature. The temperature range for the DMA analysis was extended to 210 °C to resolve the maximum of the peak better. The DSC and DMA curves are presented in Figure 7.

In the given temperature range, the glass transition temperature was not detected by the DSC because of two possible reasons. SA-based polyols are synthesized from SA whose composition, as complex natural raw material, is very heterogeneous, the polymer matrix is inhomogeneous, and the glass transition temperature can occur in a wide range. It could be in the form of several overlapping, hardly detectable steps by the corresponding DSC equipment. In addition, it is difficult to determine the glass transition temperature if it is close to the degradation temperature of the material. The DMA results confirmed both possibilities, namely the peaks in the damping factor curves were in a wide temperature range, and the maximum peak (140–180 °C) was close to the degradation temperature. The rigid PUR foam samples started to deform from ~170 °C, which resulted in uneven curves; therefore, smoothing with the adjacent-averaging method was conducted to obtain the glass transition temperature. The type of SA-based polyol and its amount in the PUR formulation affected the glass transition temperature as follows: the higher the OH value of the SA-based polyol, the higher the glass transition temperature of the corresponding rigid PUR foams, and the higher the content of the same SA-based polyol, the lower the peak in the DMA damping factor curve. 

**Figure 8:**
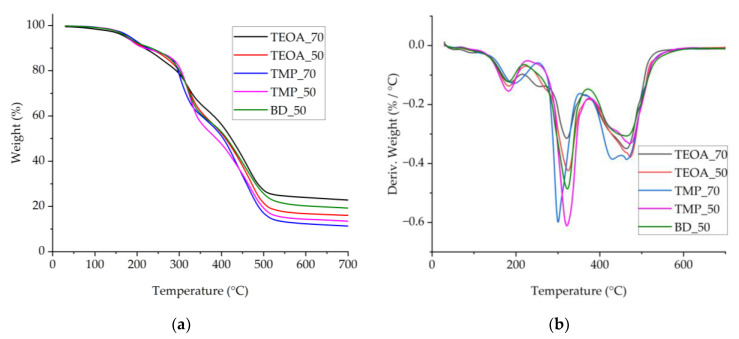
Thermogravimetric analysis: (**a**) TGA curves; (**b**) a derivative of TGA.

The TGA and DTG curves, displayed in Figure 8, show the typical degradation profile of the rigid PUR foams. In the DTG curves, three to five peaks occurred, corresponding to the three main stages in the degradation. In the first step, in the range from 150 °C to 280 °C, low molecular weight compounds evaporate, and allophanate and biuret crosslinks decompose [34]. Unlike other samples, TEOA_70 showed a peak at 250 °C, and TEOA_50 showed a shoulder peak at 270 °C, which corresponds to the degradation of carbamate in the rigid PUR foams and has been reported previously for rigid PUR foams from polyols synthesized with TEOA [34]. The main decomposition step occurred between 280 °C and 400 °C and peaked at around 320 °C, where the urethane bond is degraded [40]. The third step appears as two overlapping peaks in the range above 400 °C at a maximum of 465 °C. In addition, a peak at ~425 °C can be discerned for TMP_50 and TMP_70. In this range, the strongest bonds in the PUR backbone degrade [34]. Decomposition of suberin-like structures has been reported around the temperature of 200 °C, ~400 °C, and 450 °C [41], which coincides with the first and third degradation stages for rigid PUR foams.

The thermal stability characteristics of the developed rigid PUR foams are listed in Table 6 (first onset—thermal degradation onset temperature, T_m5%_—temperature at weight loss of 5%, T_m10%_—temperature at weight loss of 10%, Tmax_1_, Tmax_2_, Tmax_3_—DTG peaks in temperature). For all the rigid PUR foam samples, the first onset temperatures are about 155 °C in the margins of error. The visible differences in the residue value at 700 °C are discernible in Figure 8a. The lowest residues were for foams from SA_TMP polyols (residue 11.5% and 13.8%). The highest residue of 21.1% was for the TEOA_70 sample with the highest SA content. This observation agrees with the findings reported by Ferreira et al. [42], where birch outer bark and cork suberin tended to form carbonaceous solid residue after thermal degradation up to 600 °C. Thus, more SA content accounts for more solid residue formed after thermal degradation. This means that such foams have the potential to exhibit higher flammability resistance, which will be tested in future research.

#### 3.2.5. Comparison with Reference Foams 

The developed rigid PUR foams were compared with reference foams, namely other published results, which are summarized in Table 7. The results for TEOA_70 (chosen as the sample with the highest SA content) have been repeated in Table 7 to make the data comparison more convenient. 

Closed cell rigid PUR foams from renewable raw materials and commercial materials from petroleum-based polyols were chosen as the reference foams. The reference foams have been characterized with such common thermal insulation material parameters as the closed cell content, thermal conductivity, and compressive strength. The data were taken from the references shown in Table 7. NFC_0% was obtained from polyols based on tall oil fatty acids. The epoxidation and oxirane ring opening with TMP was carried out to synthesize the base polyol ETOFA_TMP, which made 85 wt.% from all the polyols into the PUR formulation [43]. This polyol is similar to the ETO_TMP polyol used in our research. The PU/LP/30 polyol component mainly consisted of the commercially used polyether-type polyol (Rokopol RF551) from which 30 wt.% was substituted with lignin-based polyol [44]. The RO/PET 1/6 base polyol was made from both a renewable part (rapeseed oil) and a recyclable part (polyethylene terephthalate). It made 75 wt.% from the amount of all the polyols in the PUR formulation [45]. SBOP 100 was prepared from commercial soybean oil-based polyol as the only polyol [30]. Elastospray 1622/6 is a commercial system based on petroleum polyol, the results of which were taken from a technical data sheet [46].

The present study has successfully developed a rigid PUR foam TEOA_70, which incorporated a significant proportion of renewable materials. In comparison to the reference foams, the developed foam TEOA_70 exhibits excellent qualities, including remarkably low thermal conductivity and one of the highest levels of compression strength. Such a low thermal conductivity for TEOA_70 could be due to the latest blowing agent Opteon^TM^ with lower vapor thermal conductivity (0.0104 W/m·K) than Solkane 365/227 (0.0109 W/m·K) or CO_2_ (0.0153 W/m·K) [47]. In addition, due to the high viscosity of SA-based polyols, smaller cells were formed which could improve the thermal insulation properties. The compressive strength of PU/LP/30 is higher because of the higher apparent density and would be approximately the same if normalized to a unified apparent density. While the exact proportion of renewable materials has not been disclosed by all authors, it is likely that TEOA_70 has one of the highest levels among the compared studies due to the exclusive use of bio-based polyols in the formulation of the PUR foam. 

## 4. Conclusions

The purpose of this study was to identify the best synthesis routes for novel SA-based polyols, obtain rigid PUR foams, and evaluate their competitiveness and potential ability as thermal insulation material. The SA from birch outer bark was used as a raw material for the synthesis of twelve polyols by esterification of acid carboxyl groups with various functionality and molecular weight alcohols, with the free carboxyl group to alcohol molar ratio of 1:1 and 1:2. Three SA-based polyols with an OH value ~410–480 mg KOH/g, an acid value lower than 15 mg KOH/g, and tolerable viscosity were chosen for the experimental obtaining of rigid PUR foam.

Five free-rise rigid PUR foam samples were successfully prepared from three pre-warmed up types of novel SA-based polyols and a tall oil-based polyol. The SA content in the PUR foams varied from 10 wt.% to 14 wt.%, and a total renewable material content of 20 wt.% was reached. The SA-based polyol synthesized with TEOA showed autocatalytic properties; therefore, it was possible to decrease the amount of catalyst in the PUR formulations with this polyol. 

The prepared SA-based rigid PUR foams demonstrated comparable mechanical and thermal properties to the reference foams in other published results. The apparent density (~33 kg/m^3^), as well as the closed cell content (~94%), compression strength (0.25 MPa, parallel to the foaming direction), and thermal conductivity (~0.019 W/(m·K)), approved the potential ability of the SA-based rigid PUR foam production as thermal insulation material. Using SA-based polyols in rigid PUR foams can contribute to a more circular economy, reduce the dependence on fossil fuels, and promote sustainability.

The interfering factor in the commercial application of SA-based polyols could be the high viscosity that required the pre-warming of the polyols. Therefore, polyol modification to lower the viscosity is suggested as a future research direction. As well as the optimization of the PUR formulations to use only SA-based polyols could be part of a future investigation. 

## Figures and Tables

**Table 1:** Parameters of the polyol synthesis.

Multifunctional Alcohol	Molar Ratio	Mass of Alcohol, g	Synthesis Temperature, °C	Acronym of Polyol
DEG	1:1	17.0	185	SA_DEG_1
	1:2	34.0		SA_DEG_2
DEOA	1:1	16.9	145	SA_DEOA_1
	1:2	33.8		SA_DEOA_2
EG	1:1	10.0	180	SA_EG_1
	1:2	20.0		SA_EG_2
BD	1:1	14.5	170	SA_BD_1
	1:2	28.9		SA_BD_2
TMP	1:1	21.5	185	SA_TMP_1
	1:2	43.0		SA_TMP_2
TEOA	1:1	23.9	175	SA_TEOA_1
	1:2	47.8		SA_TEOA_2

**Table 2:** Formulations of the rigid PUR foams.

Component		Weight, g					
		TEOA_70	TEOA_50	TMP_70	TMP_50	BD_70	BD_50
Polyols	SA_TEOA_2	70	50	-	-	-	-
	SA_TMP_2	-	-	70	50	-	-
	SA_BD_2	-	-	-	-	70	50
	ETO_TMP	30	50	30	50	30	50
Flame retardant	TCPP	30	30	30	30	30	30
Catalysts	PC CAT TKA 30	0.5	0.5	0.5	0.5	0.5	0.5
	Polycat NP10	1.2	1.2	5.0	5.0	5.0	5.0
Surfactant	L-6915	2.5	2.5	2.5	2.5	2.5	2.5
Blowing agents	Total water	2.0	2.0	2.0	2.0	2.0	2.0
	Opteon™ 1100	30	35	35	35	35	35
Isocyanate	pMDI *	166	166	170	170	157	160

* NCO/OH molar ratio was 1.2 for all the formulations.

**Table 3:** Summary of the common characteristics of synthesized SA-based polyols.

Synthesized Polyols	Acid Value, mg KOH/g	OH Value, mg KOH/g	Apparent Viscosity, mPa·s (50 s^−1^)	Moisture, %	Renewable Content, %
SA_BD_1	27	304	5.03·10^5^	<0.2	87.4
SA_BD_2	14	411	3.64·10^5^	<0.2	77.6
SA_EG_1	37	n.a.	n.a.	n.a.	90.9
SA_EG_2	26	565	8.02·10^6^	<0.2	83.4
SA_DEG_1	28	n.a.	n.a.	n.a.	85.5
SA_DEG_2	20	470	5.96·10^5^	<0.2	74.6
SA_DEOA_1	25	n.a.	n.a.	n.a.	85.6
SA_DEOA_2	22	397	7.29·10^6^	<0.2	74.8
SA_TEOA_1	24	351	6.34·10^5^	<0.2	80.7
SA_TEOA_2	13	454	4.38·10^5^	<0.2	67.6
SA_TMP_1	20	320	2,56·10^6^	<0.2	82.3
SA_TMP_2	14	478	7.27·10^5^	<0.2	69.9

**Table 4:** Rigid PUR foam morphology parameters.

	TEOA_70	TEOA_50	TMP_70	TMP_50	BD_70	BD_50
Closed cell content, vol.%	93.4 ± 0.3	93.2 ± 0.3	94.2 ± 0.3	94.4 ± 0.2	87.0 ± 0.4	95.0 ± 0.3
Average cell diameter Z, µm	235 ± 25	282 ± 19	235 ± 24	265 ± 15	n.a.	372 ± 31
Average cell diameter X, µm	192 ± 13	209 ± 16	203 ± 10	218 ± 10	n.a.	235 ± 11
Geometrical anisotropy	1.2	1.4	1.2	1.2	n.a.	1.6

**Table 5:** Summary of selected characteristics of the developed SA-based rigid PUR foams.

	TEOA_70	TEOA_50	TMP_70	TMP_50	BD_50
Foaming start time, s	30	29	53	27	44
Foam gel time, s	79	85	94	88	56
Tack-free time, s	127	135	142	113	84
Foam rise time, s	112	107	174	97	140
Shrinkage, %	1.7	0.8	6.2	2.3	15.0
SA content, %	14.3	10.0	14.1	10.1	11.6
Total renewable material content, %	20.6	20.0	20.2	19.9	21.6
Apparent density, kg/m^3^	35.1 ± 0.5	31.7 ± 1.0	35.0 ± 0.4	33.9 ± 0.7	33.4 ± 0.4
Compressive strength Z, MPa	0.253 ± 0.012	0.237 ± 0.006	0.261 ± 0.017	0.265 ± 0.013	0.239 ± 0.008
Compressive modulus Z, MPa	6.52 ± 0.36	5.40 ± 0.10	6.42 ± 0.18	6.60 ± 0.20	5.50 ± 0.20
Thermal conductivity ± 0.0002, W/(m·K)	0.0192	0.0193	0.0190	0.0189	0.0194

**Table 6:** Thermal properties of the rigid PUR foam samples.

Sample	First Onset ^1^, °C	T_m5%_^2^, °C	T_m10%_^2^, °C	Residue ^3^, %	T_max1_^4^, °C	T_max2_^4^, °C	T_max3_^4^, °C
TEOA_70 ^5^	159.6 ± 3.6	176.5 ± 3.9	218.8 ± 2.3	21.1 ± 1.5	188.6 ± 2.0	323.1 ± 2.0	464.3 ± 0.9
TEOA_50	154.6 ± 4.0	177.7 ± 3.5	224.5 ± 1.0	16.2 ± 0.2	183.6 ± 3.5	325.4 ± 3.3	472.0 ± 1.2
TMP_70 ^6^	156.0 ± 9.7	181.4 ± 4.1	225.2 ± 1.4	11.5 ± 0.3	194.1 ± 1.1	300.5 ± 1.2	464.8 ± 3.0
TMP_50 ^6^	155.2 ± 6.8	176.8 ± 5.4	219.7 ± 2.7	13.8 ± 0.3	184.1 ± 6.1	322.7 ± 4.1	471.0 ± 4.5
BD_50	150.8 ± 5.1	181.1 ± 5.1	236.0 ± 2.6	19.3 ± 0.2	185.0 ± 4.6	325.5 ± 3.6	464.8 ± 1.7

^1^ Thermal degradation onset temperature. ^2^ Temperature at a weight loss of 5% and 10%. ^3^ Weight of the solid residue remaining at 700 °C. ^4^ DTG peaks in temperature. ^5^ For TEOA_70 an additional peak appears at 250.4 ± 4.4 °C. ^6^ For TMP_70 and TMP_50 an additional peak appears at 429.1 ± 2.2 °C and 425.4 ± 1.3 °C.

**Table 7:** TEOA_70 and reference rigid PUR foam characteristics.

	TEOA_70	NFC_0% [43]	PU/LP/30 [44]	RO/PET 1/6 [45]	SBOP 100 [30]	Elastospray 1622/6 [46]
Main blowing agent	Opteon™ 1100	c-pentane	water	Solkane 365/227	n-pentane	HFC ^1^
Total renewable material content, %	20.6	18.1	n.a.	4.5 ^2^	n.a.	n.a.
Apparent density, kg/m^3^	35.1	35.0	41.4	45.0	46.4	37.0
Closed cell content, vol.%	93	96	86	95	91	95
Thermal conductivity, W/(m·K)	0.0192	0.022	0.0229	0.0206	0.0242	0.0205
Compressive strength ^3^, MPa	0.25	0.20	0.34	0.25	0.17	0.22

^1^ Hydrofluorocarbon. ^2^ Calculated using data available in publication [45]. ^3^ Parallel to the foam rise direction.

## Data Availability

Not applicable.

## References

[1] Backes J.G., Traverso M. (2022). Life Cycle Sustainability Assessment as a Metrics towards SDGs Agenda 2030. Curr. Opin. Green Sustain. Chem..

[2] Ma Y., Xiao Y., Zhao Y., Bei Y., Hu L., Zhou Y., Jia P. (2022). Biomass Based Polyols and Biomass Based Polyurethane Materials as a Route towards Sustainability. React. Funct. Polym..

[3] Tuladhar R., Yin S. (2019). Sustainability of Using Recycled Plastic Fiber in Concrete. Use of Recycled Plastics in Eco-Efficient Concrete.

[4] Simón D., Borreguero A.M., de Lucas A., Gutiérrez C., Rodríguez J.F., Jiménez E., Cabañas B., Lefebvre G. (2015). Sustainable Polyurethanes: Chemical Recycling to Get It. Environment, Energy and Climate Change I Environmental Chemistry of Pollutants and Wastes.

[5] Msanne J., Kim H., Cahoon E.B. (2020). Biotechnology Tools and Applications for Development of Oilseed Crops with Healthy Vegetable Oils. Biochimie.

[6] Ranjbar S., Malcata F.X. (2022). Challenges and Prospects for Sustainable Microalga-Based Oil: A Comprehensive Review, with a Focus on Metabolic and Genetic Engineering. Fuel.

[7] Vevere L., Fridrihsone A., Kirpluks M., Cabulis U. (2020). A Review of Wood Biomass-Based Fatty Acids and Rosin Acids Use in Polymeric Materials. Polymers.

[8] Rizikovs J., Godina D., Makars R., Paze A., Abolins A., Fridrihsone A., Meile K., Kirpluks M. (2021). Suberinic Acids as a Potential Feedstock for Polyol Synthesis: Separation and Characterization. Polymers.

[9] Rizhikovs J., Zandersons J., Dobele G., Paze A. (2015). Isolation of Triterpene-Rich Extracts from Outer Birch Bark by Hot Water and Alkaline Pre-Treatment or the Appropriate Choice of Solvents. Ind. Crops Prod..

[10] Gandini A., Pascoal Neto C., Silvestre A.J.D. (2006). Suberin: A Promising Renewable Resource for Novel Macromolecular Materials. Prog. Polym. Sci..

[11] Godina D., Makars R., Paze A., Rizhikovs J. (2023). Analytical Method Cluster Development for Comprehensive Characterisation of Suberinic Acids Derived from Birch Outer Bark. Molecules.

[12] Rižikovs J., Zandersons J., Paže A., Tardenaka A., Spince B. (2014). Isolation of Suberinic Acids from Extracted Outer Birch Bark Depending on the Application Purposes. Balt. For..

[13] Abolins A., Pomilovskis R., Vanags E., Mierina I., Michalowski S., Fridrihsone A., Kirpluks M. (2021). Impact of Different Epoxidation Approaches of Tall Oil Fatty Acids on Rigid Polyurethane Foam Thermal Insulation. Materials.

[14] Pomilovskis R., Mierina I., Beneš H., Trhlíková O., Abolins A., Fridrihsone A., Kirpluks M. (2022). The Synthesis of Bio-Based Michael Donors from Tall Oil Fatty Acids for Polymer Development. Polymers.

[15] (2000). Plastics (polyester resins) and Paints and Varnishes (binders)—Determination of Partial Acid Value and Total Acid Value.

[16] (2016). Binders for Paints and Varnishes—Determination of Hydroxyl Value—Part 2: Titrimetric Method Using a catalyst.

[17] (2006). Cellular Plastics and Rubbers—Determination of Apparent Density.

[18] (1991). Thermal Insulation—Determination of Steady-State Thermal Resistance and Related Properties—Heat Flow Meter Apparatus.

[19] (2016). Rigid Cellular Plastics—Determination of the Volume Percentage of Open Cells and of Closed Cells.

[20] (2020). Standard Test Method for Cell Size of Rigid Cellular Plastics.

[21] (2021). Rigid cellular Plastics—Determination of Compression Properties.

[22] Hawkins M.C., O’Toole B., Jackovich D. (2005). Cell Morphology and Mechanical Properties of Rigid Polyurethane Foam. J. Cell. Plast..

[23] Kirpluks M., Vanags E., Abolins A., Michalowski S., Fridrihsone A., Cabulis U. (2020). High Functionality Bio-Polyols from Tall Oil and Rigid Polyurethane Foams Formulated Solely Using Bio-Polyols. Materials.

[24] Yakushin V., Stirna U., Bikovens O., Misane M., Sevastyanova I., Vilsone D. (2013). Synthesis and Characterization of Novel Polyurethanes Based on Tall Oil. Mater. Sci..

[25] Fridrihsone-Girone A., Stirna U. (2014). Characterization of Polyurethane Networks Based on Rapeseed Oil Derived Polyol. Polimery.

[26] Yakushin V., Stirna U., Bikovens O., Misane M., Sevastyanova I., Vilsone D. (2014). Synthesis and Characterization of Novel Polyurethanes Basedon Vegetable Oils Amide and Ester Polyols. Mater. Sci..

[27] Zieleniewska M., Leszczynski M.K., Kuranska M., Prociak A., Szczepkowski L., Krzyzowska M., Ryszkowska J. (2015). Preparation and Characterisation of Rigid Polyurethane Foams Using a Rapeseed Oil-Based Polyol. Ind. Crops Prod..

[28] Prociak A., Kurañska M., Malewska E., Szczepkowski L., Zieleniewska M., Ryszkowska J., Ficon J., Rzasa A. (2015). Biobased Polyurethane Foams Modified with Natural Fillers. Polimery.

[29] Kairytė A., Vėjelis S. (2015). Evaluation of Forming Mixture Composition Impact on Properties of Water Blown Rigid Polyurethane (PUR) Foam from Rapeseed Oil Polyol. Ind. Crops Prod..

[30] Tan S., Abraham T., Ference D., MacOsko C.W. (2011). Rigid Polyurethane Foams from a Soybean Oil-Based Polyol. Polymer (Guildf.).

[31] Thirumal M., Khastgir D., Singha N.K., Manjunath B.S., Naik Y.P. (2008). Effect of Foam Density on the Properties of Water Blown Rigid Polyurethane Foam. J. Appl. Polym. Sci..

[32] Hejna A., Kirpluks M., Kosmela P., Cabulis U., Haponiuk J., Piszczyk Ł. (2017). The Influence of Crude Glycerol and Castor Oil-Based Polyol on the Structure and Performance of Rigid Polyurethane-Polyisocyanurate Foams. Ind. Crops Prod..

[33] Ivdre A., Abolins A., Sevastyanova I., Kirpluks M., Cabulis U., Merijs-Meri R. (2020). Rigid Polyurethane Foams with Various Isocyanate Indices Based on Polyols from Rapeseed Oil and Waste PET. Polymers.

[34] Paberza A., Fridrihsone-Girone A., Abolins A., Cabulis U. (2015). Polyols from Recycled Poly (Ethylene Terephthalate) Flakes and Rapeseed Oil for Polyurethane Foams. Polimery.

[35] Thermal Insulation Materials Made of Rigid Polyurethane Foam. https://highperformanceinsulation.eu/wp-content/uploads/2016/08/Thermal_insulation_materials_made_of_rigid_polyurethane_foam.pdf.

[36] Rigid Polyurethane Foams as an Insulation Material. https://www.ufoam.com/images/ufoam/downloads/brochures-tech-info/technical-literature4-rigid-pu-foam-as-insulation.pdf.

[37] Ahern A., Verbist G., Weaire D., Phelan R., Fleurent H. (2005). The Conductivity of Foams: A Generalisation of the Electrical to the Thermal Case. Colloids Surfaces A Physicochem. Eng. Asp..

[38] Andersons J., Kirpluks M., Stiebra L., Cabulis U. (2016). Anisotropy of the Stiffness and Strength of Rigid Low-Density Closed-Cell Polyisocyanurate Foams. Mater. Des..

[39] Paberza A., Stiebra L., Cabulis U. (2015). Photodegradation of Polyurethane Foam Obtained from Renewable Resource–Pulp Production Byproducts. J. Renew. Mater..

[40] Jabar J.M. (2022). Production of Sustainable Rigid Polyurethane Foam from Chemically Modified Underutilized Jatropha Curcas L Seed Oil: Influence of Polyol Chemical Structure on Properties of Polymer. Curr. Res. Green Sustain. Chem..

[41] Makars R., Rizikovs J., Godina D., Paze A., Merijs-Meri R. (2022). Utilization of Suberinic Acids Containing Residue as an Adhesive for Particle Boards. Polymers.

[42] Ferreira R., Garcia H., Sousa A.F., Freire C.S.R., Silvestre A.J.D., Rebelo L.P.N., Silva Pereira C. (2013). Isolation of Suberin from Birch Outer Bark and Cork Using Ionic Liquids: A New Source of Macromonomers. Ind. Crops Prod..

[43] Kirpluks M., Ivdre A., Fridrihsone A., Cabulis U. (2020). Tall Oil Based Rigid Polyurethane Foams Thermal Insulation Filled with Nanofibrillated Cellulose. Polimery.

[44] Kurańska M., Pinto J.A., Salach K., Barreiro M.F., Prociak A. (2020). Synthesis of Thermal Insulating Polyurethane Foams from Lignin and Rapeseed Based Polyols: A Comparative Study. Ind. Crops Prod..

[45] Ivdre A., Fridrihsone-Girone A., Abolins A., Cabulis U. (2018). Effect of Different Concentration of Rapeseed Oil and Recycled Poly (Ethylene Terephthalate) in Polyols for Rigid Polyurethane Foams. J. Cell. Plast..

[46] BASF Technical Data Sheet Elastospray 1622/6. https://www.torkret.by/files/files/Elastospray1622-6.pdf.

[47] Randall D., Lee S. (2002). The Polyurethanes Book.

